# Research on abnormal pressure of dark shale in the Tiemulike formation of the Yining Sag, Ili basin

**DOI:** 10.1038/s41598-026-36584-1

**Published:** 2026-01-28

**Authors:** Weihua Yang, Yan Ren, Metenbaev Igor Igorevich

**Affiliations:** 1 School of Geography and Tourism, Nanyang Normal University, Nanyang, 473061 China; 2 Henan Province Resource and Environment Spatiotemporal Big Data Development Innovation Laboratory , Nanyang Normal University, Nanyang, 473061 China; 3 Nanyang City Key Laboratory of Dushan Jade, Nanyang Normal University, Nanyang, 473061 China

**Keywords:** Shale, Tiemulike Formation, Abnormal pressure, Equilibrium depth method, Ili Basin, Energy science and technology, Solid Earth sciences

## Abstract

The study of abnormal pressure in the dark shale of the Tiemulike Formation in the Yining Sag of the Ili Basin holds significant importance for its oil and gas exploration. Based on the Acoustic time difference data of shale, the overexposure development interval in the Tiemulike Formation was of the Yining Sag determined. The equilibrium depth method had been adopted to quantitatively calculate the excess pressure and pressure coefficient of dark shale in the Tiemulike Formation for the first time. The distribution map of abnormal excess pressure in single well profiles and well-tie comparison profile had been drawn, and the abnormal excess pressure characteristics of the shale had been fully Analyzed. The results show that the pressure coefficient of the dark shale in Tiemulike Formation is 1.21–1.95, belonging to the weak-high pressure to Ultra-high pressure layers. The excess pressure gradually increased from the top to the deeper layers in the Tiemulike Formation, with three abnormal pressure significantly increased layers. The abnormal excess pressure in Tiemulike Formation is about 5–10 MPa higher than that in the overlying Upper Permian Basiergan Formation, indicating high abnormal pressure characteristics. The horizontal distribution characteristics of abnormal excess pressure in Tiemulike Formation show that the abnormal excess pressure is mostly above 10 MPa, with a highest reaching 29.4 MPa. The Higher abnormal excess pressure has good continuity in the horizontal direction, several high abnormal fluid pressure compartments were formed in the central low-laying area of Yining Sag. The formation of abnormal excess pressure in the shale of the Tiemulike Formation in the Yining Sag was mainly controlled by the hydrocarbon generation expansion of dark shale, as well as the influence of clay mineral diagenesis.

## Introduction

Dickinson (1953) conducted a study on overpressure in the Gulf Coast region of the United States^1^, which started a new era on the study of abnormal pressure. The study of abnormal pressure is always one of the hot and difficult issues in oil and gas geological exploration, abnormal high pressure or overpressure is widely distributed in petroliferous basins, which is closely related to the formation of oil and gas reservoirs^2–4^. According to incomplete statistics, the overpressure have developed in nearly two-thirds of global sedimentary basins^5–8^. The phenomenon of overpressure developed in shale accounts for a large proportion, such as Barnett shale and Haynesville shale in North America^9^, the shale in Upper Jurassic Dingo Formation of the North Carnarvon Basin in the Northwest Shelf of Australian^10^, Zhangjiatan shale in the 7th section of the Yanchang Formation in the Ordos Basin^11^, shale in Middle Jurassic in Shengbei Sub–sag, Turpan–Hami Basin^12^, shale in Silurian Longmaxi Formation of Sichuan Basin^13,14^, shale in Middle Permian Lucaogou Formation in the southern margin of the Junggar Basin^15,16^, and shale in Middle Permian Tiemulike Formation of Ili Basin^17^, etc.

Ili Basin is one of the hot areas for oil and gas geological surveys in China, the Yining Sag is located in the eastern part of the basin, with good hydrocarbon generation potential in the dark shale of the Tiemulike Formation^18^. Several oil and gas shows had been discovered in shale of the Tiemulike Formation, revealing promising prospects for shale oil and gas exploration^19,20^. The dark shale in the Tiemulike Formation is widely distributed and thick in the Yining Sag, which is a high-quality source rock. As we all known, the overpressure in shale always play an important part in the formation of shale gas reservoir. But so far, there has been no research on the identification of overpressure and quantitative calculation of excess pressure in the shale of the Tiemulike Formation in the Yining Sag. The research in domestic and foreign has confirmed that the study of shale overpressure has practical application value in the exploration and development of shale gas reservoirs, improving recovery rates, and optimizing development strategies. Therefore, it is urgent to carry out systematic research on the overpressure of the Tiemulike Formation in the Yining Depression.

### Geological setting

**Fig. 1:**
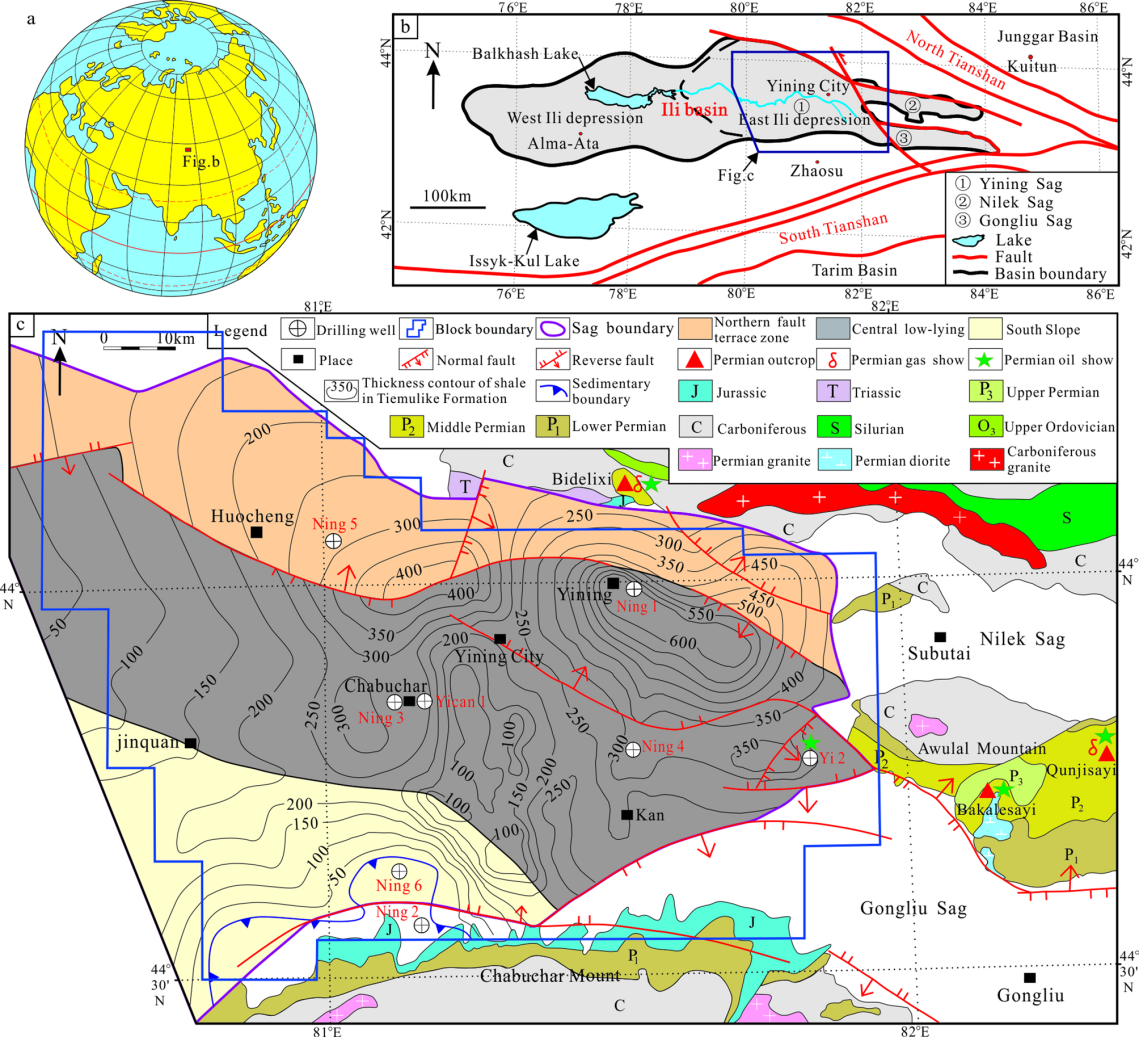
Comprehensive geological map and thickness contour of dark mud shale in Tiemulike Formation of Yining Sag (revised after^17,23^) (this figure is generated in CorelDRAW 2018 software, https://www.coreldrawchina.com/).

The Ili Basin is located at China-Kazakhstan Border, which Composed of East Ili Depression and West Ili Depression^21–23^ (Fig. 1). The Yining Sag is in the Middle of East Ili Depression, with a ladder shape, which has an area of 9238 km^2^. There are three substructural units in the Yining Sag: the Northern fault terrace zone, Central Low-lying zone, and Southern Slope. The main exploration and development target is the dark shale in the Tiemulike Formation, which developed in the Central Low-lying of the Yining Sag.

**Fig. 2:**
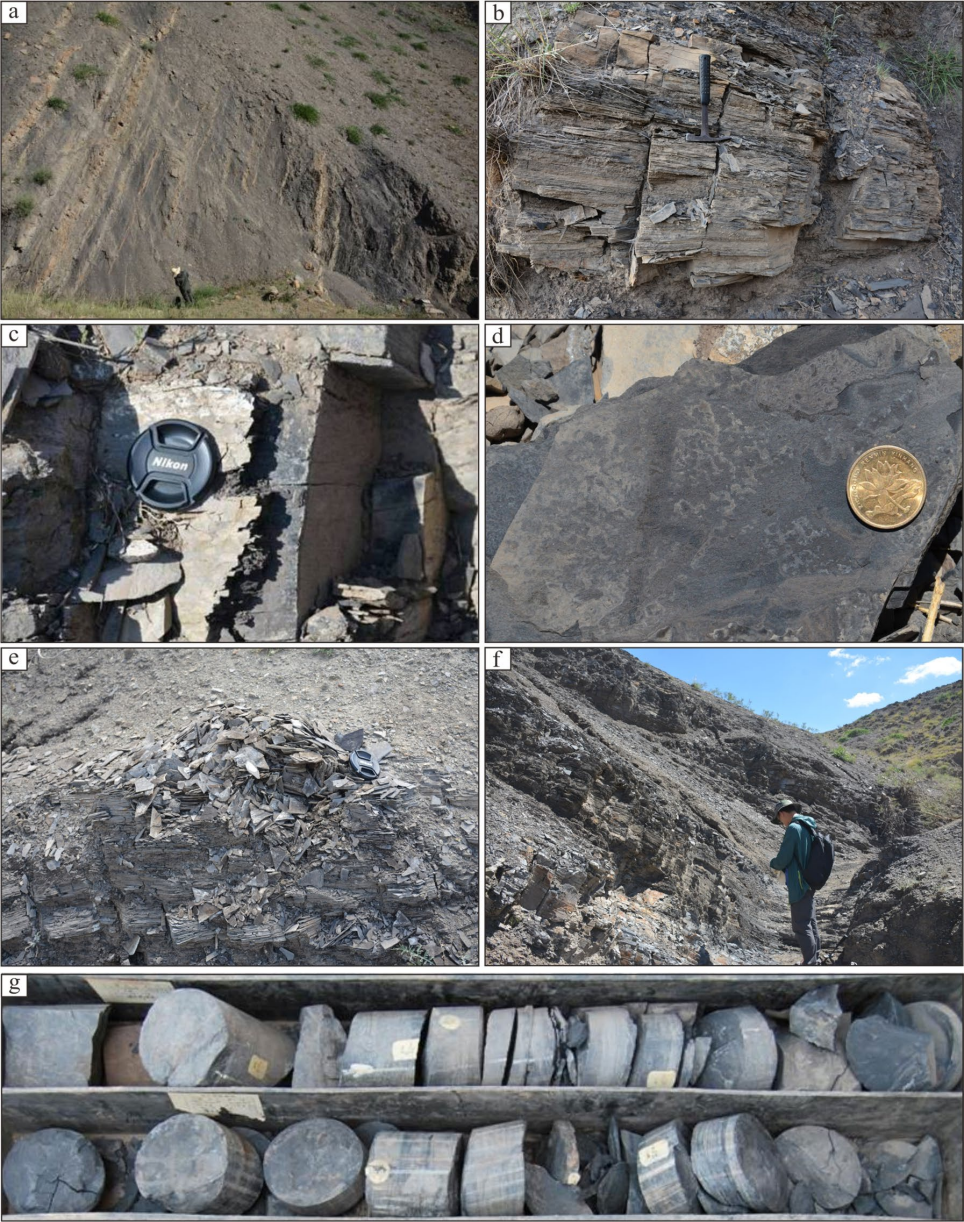
Photos of shale in Tiemulike Formation in Yining Sag. (**a**) Thick shale interbedded with fine sandstone in the lower part of the Tiemulike Formation at Qunjisayi; (**b**) Grey black shale in the lower part of the Tiemulike Formation at Qunjisayi; (**c**) Calcareous shale in the lower part of the Tiemulike Formation at Bskalesayi, the asphalt veins is visible; (**d**) Black shale in the lower part of the Tiemulike Formation at Bskalesayi; (**e**) The bedding of shale developed in Tiemulike Formation at Bedelixi; (**f**) Calcareous shale in the lower part of the Tiemulike Formation at Bedelixi; (**g**) Gray black shale in the lower part of the Tiemulike Formation in Ning 3 well, the horizontal bedding is visible.

The Tiemulike Formation is mainly composed of terrestrial lake sediments. There are thick gray conglomerate, conglomerate sandstone, and dark gray fine sandstone in the lower section, with thin layers of coal seams/carbonaceous mudstone locally developed. There are dark shale, mudstone, calcareous shale inter-bedded with thin layered limestone and fine sandstone in the middle section, which is the main development interval of dark shale, with thickness mostly between 150-600 m and actively oil and gas shows (Fig. 2). There are gray-yellow green feldspar sandstone, fine sandstone interbedded with medium thick layered tuffaceous conglomerate and conglomerate lens in the upper section, the plant fragments and fossilized stems are visible.

Up to now, there are only four drilling wells encountered the Tiemulike Formation in Yining Sag, such as Yican 1 well, Yi 2 well, Ning 3 well and Ning 4well. None of the wells drilled through the Tiemulike Formation, with encountering thicknesses of 403.9 m, 560 m, 372 m, and 1242.3 m respectively. The lithology is mainly composed of dark shale, gray calcareous shale, mudstone, silty limestone inter-bedded with sandstone and conglomerate. There are multiple weak fluorescent shows observed in shale of the Tiemulike Formation in Yican 1. There is an oil spot was observed in the crack of the dark gray calcareous shale of the Tiemulike Formation at 1443.8–1445.8 m in Yi 2 well, with multiple weak fluorescence, but oil and gas testing has not been conducted.

### Samples and methods

#### Samples

References related to the Yining region had been reviewed and analyzed. Logging data of four drilling wells were collected, including the Yican 1 well and Yi 2 well drilled by Xinjiang Petroleum Administration Bureau and the Ning 3 well and Ning 4 well drilled by Zhongyuan Petroleum Exploration Bureau. Field geological survey has been conducted on the dark mudstone outcrops of the Tiemulike Formation around the Yining Sag, covering a distance of 7.8 km, including the Bidelixi section at the north of Yining, the Qunjisayi section and the Bakalesayi section at the west of Yining Sag, taking 237 photos, drawing 35 geological sketches. The 372 m core of the Tiemulike Formation in Ning 3 well had been observed, taking 32 photos. The Compaction trend line fitted to the relationship between acoustic time difference and burial depth in Yining Sag had been made. 3 excess pressure profiles of single well and 1 connected well comparison profile of abnormal excess pressure were drawn.

## Methods

### Research technology route

The equilibrium depth method, also known as equivalent depth method, was firstly proposed by Makara in 1968^24^, which has been translated and introduced into China by Chen Heli in 1981^25^. Chen H. & Luo X. (1988) found that the compaction effect of sediment in the target layer is irreversible at the maximum buried depth, therefore, the fluid pressure calculated by the equilibrium depth method reflects the distribution of layer pressure at its maximum burial depth^26,27^.

First of all, the regional geology of Yining area, oil and gas discoveries of Yining Sag, the logging curve data of all drillings encountered with the Tiemulike Formation in the Yining Sag had been collected and analyzed. Next, the compaction trend line of the relationship between acoustic time differences with burial depth in Yinng Sag had been fitted. According to the acoustic time difference data at the turning point of shale, the equilibrium depth method had been adopted, the excess pressure and pressure coefficient of dark shale in the Tiemulike Formation were quantitatively calculated for the first time. Then, the distribution map of abnormal excess pressure in single well profiles had been made, so the vertical variation characteristics of excess pressure had been analyzed. The well-tie comparison profile of abnormal excess pressure had been drawn, and the horizontal distribution characteristics of abnormal excess pressure of Tiemulike Formation had been systematically studied. Finally, the main controlling factors of abnormal excess pressure developed in the shale of the Tiemulike Formation had been discussed.

## Research steps

In this study, the equilibrium depth method was used to calculate the abnormal pressure of the Permian Tiemulike Formation in the Yining Sag. The specific steps are as follows.

Firstly, the acoustic time difference values of shale layers with different burial depths on the logging curve has been read, the relationship curve between acoustic time difference and burial depth has been created. To eliminate interference, the pure shale sections with the single layer thickness greater than 2 m and no obvious expansion or high natural gamma in logging curves were chose to read acoustic time difference data. In this paper, the acoustic time difference and corresponding depth data of shale from Yican 1 well, Ning 3 well, Ning 4 well, and Yi 2 well were counted.

**Fig. 3:**
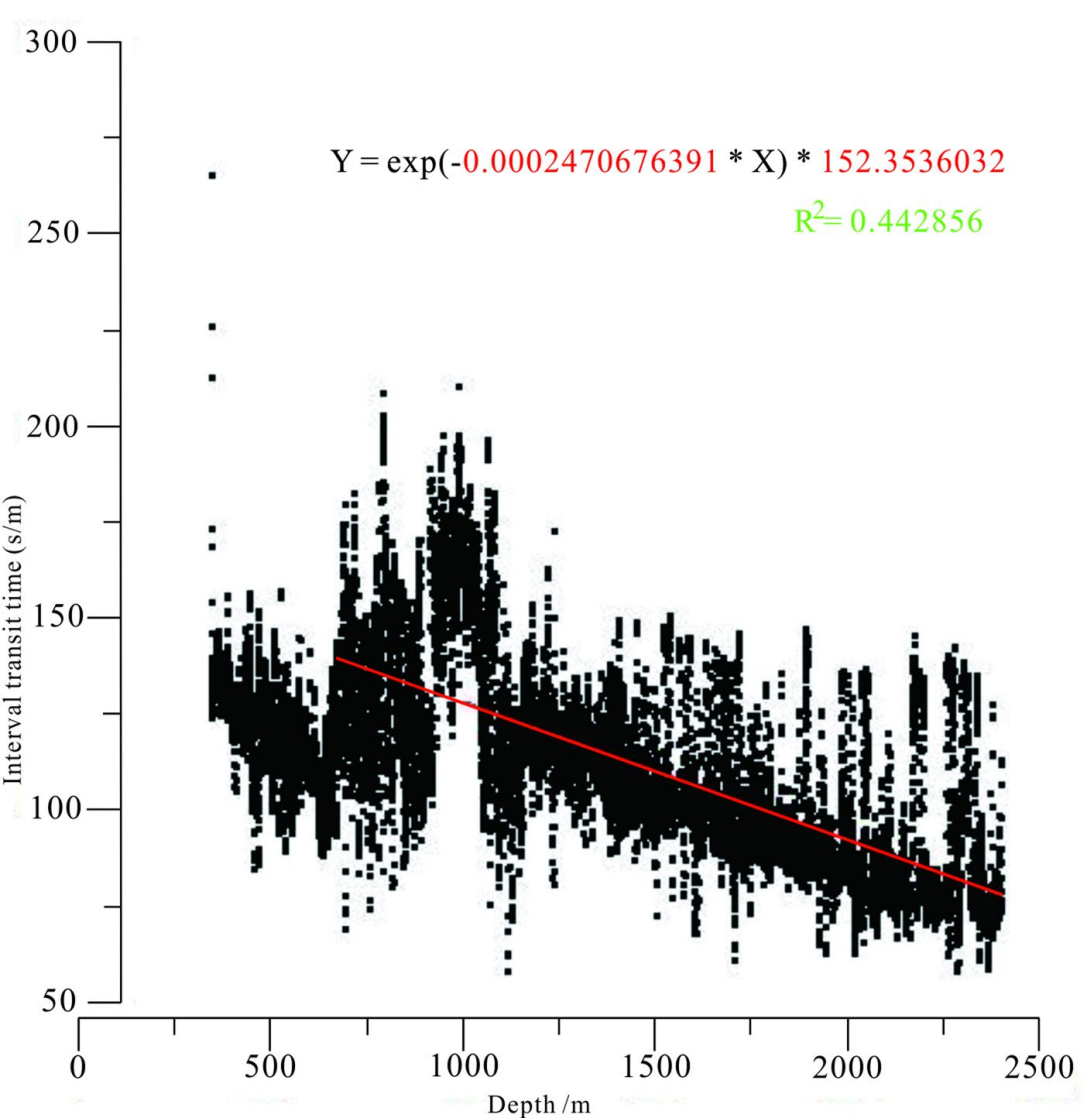
Compaction trend line fitted to the relationship between acoustic time difference and burial depth in Yinng Sag (this figure is generated in CorelDRAW 2018 software, https://www.coreldrawchina.com/).

Secondly, according to the relationship between acoustic time difference (AC) and burial depth from Yican 1 well, Ning 3 well, Ning 4 well, and Yi 2 well, a compaction trend line had been fitted and drawn. The compaction coefficient (C) value and the original surface acoustic time difference (△*t*_*0*_) of normally compacted shale under normal compaction conditions were calculated, the value of compaction coefficient is 0.0002470676391, and the value of △*t*_*0*_ is 152.3536032. The residual sum of squares R^2^ of the fitted curve is 0.442856, which had achieved good fitting goodness (Fig. 3).

**Fig. 4:**
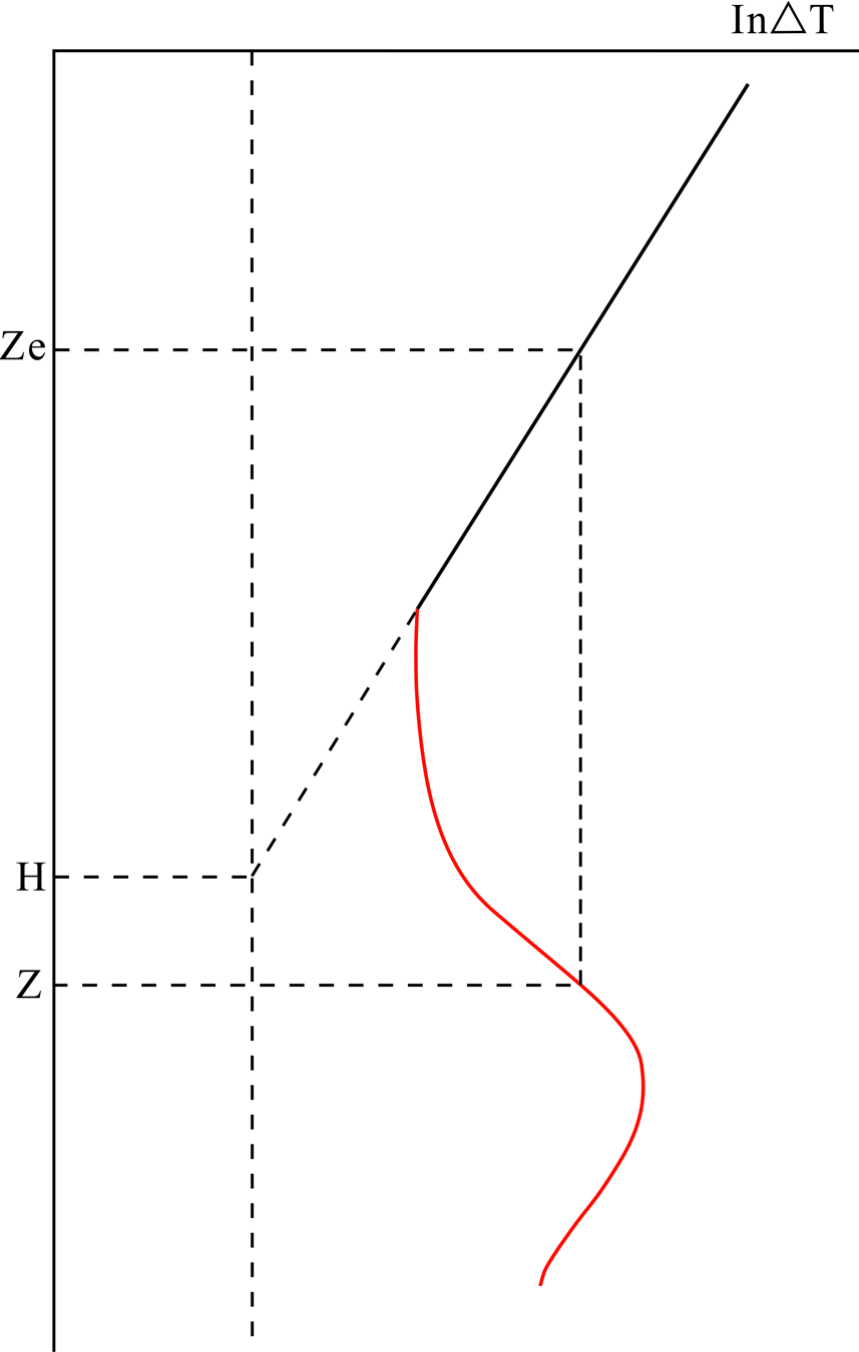
Sketch map of equilibrium depth method(this figure is generated in CorelDRAW 2018 software, https://www.coreldrawchina.com/).

Thirdly, on Relationship diagram between acoustic time difference and burial depth, the normal compaction section appears as a straight line. Assuming that Z and Ze respectively represent the depths corresponding to a point in the abnormal compaction section and a point in the normal compaction section with the same acoustic time difference of shale on the compaction curve, the porosity corresponding to these two points is equal, Ze is called the equilibrium depth of Z, also called equivalent depth (Fig. 4).

Based on the above analysis, the pore pressure of shale is expressed as Eq. (1).

1 $$P_{z} = P_{e} + \left( {S_{z} - S_{e} } \right) = \rho_{r} gZ - \left( {\rho_{r} - \rho_{w} } \right)gZ_{e}$$

The relationship between acoustic time difference and buried depth is shown as Eq. (2).

2 $$\Delta t_{e} = \Delta t_{0}^{\prime } \cdot e^{ - CZe}$$

Taking the logarithm on both sides of the above Eq. (2), we got (3) & (4).

3 $$\ln \Delta t = - CZ_{e} \ln e + \ln \Delta t_{0}$$

4 $$Z_{e} = - C^{ - 1} \left( {\ln \Delta t - \ln \Delta t_{0} } \right)$$

Finally, the abnormal pressure values in shale had been calculated. The Eq. (4) had been substituted into Eq. (1) to obtain the relationship shown in Eq. (5).

5 $$P_{{\mathrm{z}}} = \rho_{{\mathrm{r}}} gZ + \frac{{(\rho_{r} - \rho_{w} )g}}{C}\ln \frac{\Delta t}{{\Delta t_{0} }}$$

6 $$\Delta P = P_{z} - P_{e}$$

7 $$P_{c} = P_{z} /P_{e}$$

The value of gravity acceleration (g) in this calculation is 9.8 m/s^2^. Based on Zhong (2011)^26^, the rock density (*ρ*_r_) of shale layer is 2.30 g/cm^3^. The value of compaction coefficient (C) is 0.00025, and the value of △*t*_*0*_ is 152.35. Using Eq. (5), the pore pressure values of shale in the Tiemulike Formation were systematically calculated. Using Eq. (6), the values of abnormal excess pressure were calculated. Using Eq. (7), the values of pressure coefficient were calculated. The meaning of each symbol and its unit in above equations are shown in Table 1. There is more or less potential uncertainty due to stratigraphic gaps or resolution limits of log-derived pressure.

**Table 1:** Parameters of formula for calculating abnormal pressure in shale of Tiemulike Formation.

Symbol	Meaning	Unit	Symbol	Meaning	Unit
Z	Buried depth of shale	m	Z_e_	Equilibrium buried depth of shale	m
*P*_z_	Pore pressure of shale	Pa	*P*_e_	Hydrostatic pressure at equilibrium depth	Pa
S_z_	Overburden pressure at depth of Z	Pa	S_e_	Overburden pressure at equilibrium depth	Pa
*Ρ*_w_	Density of pore water in layers	kg/m^3^	*ρ*_r_	Average density of layers	kg/m^3^
△*t*	Interval transit time value of shale	µm	△*t*_*0*_	Original surface acoustic time difference	µm
C	Compaction coefficient of shale	m^-1^	g	Gravity acceleration	m/s^2^
△*P*	Excess pressure	Pa	*P*_c_	Pressure coefficient	

## Result

### Classification of abnormal pressure types

Currently, it is common to classify the abnormal pressure of layers using the pressure coefficient (Pc). Using Eq. (7), the values of pressure coefficient of shale in Tiemulike Formation in Yican 1, Ning 3 and Ning 4 were separately calculated (Tables 2, 3 and 4), which respectively are 1.38 ~ 1.50, 1.23 ~ 1.48, and 1.21 ~ 1.95. According to conventional classifications of overpressure, which was typically defined as follows: mild (1.0–1.2), moderate (1.2–1.5), high (1.5–1.8), and very high (> 1.8)^28,29^. Referencing the standardized classification system from Bowers (1995)^28^ and Zhang (2011)^29^, the pressure coefficient of the dark shale in Tiemulike Formation is mainly the moderate pressure, the high and very high pressure in same local areas.

**Table 2:** Calculation data statistics of abnormal pressure in Yican 1 well.

Stratum	Buried depth	Acoustic time difference	Interval distance	Ze /m	Pz /MPa	Pe /MPa	△*P*	Pc	classifications of overpressure
Basiergan Formation P_3_b	3519	79	153	2658.20	46.6	35.9	10.71	1.30	moderate
	3672	80	94	2607.29	50.7	37.4	13.25	1.35	moderate
	3766	77	56	2761.99	50.9	38.4	12.50	1.33	moderate
	3822	75	168	2868.51	50.8	39.0	11.87	1.30	moderate
	3990	74	50	2922.84	53.9	40.7	13.28	1.33	moderate
	4040	75	75	2868.51	55.8	41.2	14.58	1.35	moderate
	4115	75	55	2868.51	57.5	41.9	15.51	1.37	moderate
	4170	80	65	2607.29	62.0	42.5	19.45	1.46	moderate
	4235	74	80	2922.84	59.5	43.2	16.33	1.38	moderate
Tiemulike Formation P_2_t	4315	76	192	2814.90	62.6	44.0	18.67	1.42	moderate
	4507	76	113	2814.90	67.0	45.9	21.06	1.46	moderate
	4620	70	28	3147.76	65.4	47.1	18.32	1.39	moderate
	4648	74	37	2922.84	68.8	47.4	21.47	1.45	moderate
	4685	74	40	2922.84	69.7	47.7	21.93	1.46	moderate
	4725	68	50	3265.08	66.3	48.2	18.17	1.38	moderate
	4775	73	37	2977.91	71.0	48.7	22.37	1.46	moderate
	4812	75	33	2868.51	73.2	49.0	24.19	1.49	moderate
	4845	75	40	2868.51	74.0	49.4	24.60	1.50	moderate
	4885	67	25	3325.05	69.2	49.8	19.42	1.39	moderate
	4910	67	75	3325.05	69.8	50.0	19.73	1.39	moderate
	4985	70	-	3147.76	73.7	50.8	22.87	1.45	moderate

**Table 3:** Calculation data statistics of abnormal pressure in Ning 3 well.

Stratum	Buried depth	Acoustic time difference	Interval distance	Ze /m	Pz /MPa	Pe /MPa	△*P*	Pc	classifications of overpressure
Basiergan Formation P_3_b	4016	75	16	2868.51	55.2	40.9	14.28	1.35	moderate
	4032	80	14	2607.29	58.8	41.1	17.73	1.43	moderate
	4046	68	38	3265.08	51.0	41.2	9.72	1.24	moderate
	4084	85	31	2361.92	63.1	41.6	21.43	1.51	high
	4115	76	18	2814.90	58.1	41.9	16.18	1.39	moderate
Tiemulike Formation P_2_t	4133	67	15	3325.05	52.2	42.1	10.06	1.24	moderate
	4148	82	14	2507.35	62.7	42.3	20.42	1.48	moderate
	4162	68	26	3265.08	53.6	42.4	11.16	1.26	moderate
	4188	69	16	3206.00	54.9	42.7	12.22	1.29	moderate
	4204	66	19	3385.91	53.0	42.8	10.18	1.24	moderate
	4223	69	27	3206.00	55.7	43.0	12.66	1.29	moderate
	4250	71	33	3090.35	57.7	43.3	14.43	1.33	moderate
	4283	70	30	3147.76	57.8	43.7	14.13	1.32	moderate
	4313	70	19	3147.76	58.5	44.0	14.50	1.33	moderate
	4332	75	35	2868.51	62.4	44.2	18.21	1.41	moderate
	4367	72	23	3033.72	61.1	44.5	16.59	1.37	moderate
	4390	68	23	3265.08	58.7	44.7	14.00	1.31	moderate
	4413	63	32	3574.20	55.4	45.0	10.44	1.23	moderate
	4445	65	30	3447.71	57.7	45.3	12.41	1.27	moderate
	4475	65	-	3447.71	58.3	45.6	12.69	1.28	moderate

**Table 4:** Calculation data statistics of abnormal pressure in Ning 4 well.

Stratum	Buried depth	acoustic time difference	Interval distance	Ze /m	Pz /MPa	Pe /MPa	△*P*	Pc	classifications of overpressure
Basiergan Formation P_3_b	2405	77	25	2719.09	20.6	24.5	-3.9	0.84	–
	2430	83.5	20	2391.08	25.3	24.8	0.5	1.02	mild
	2450	85	30	2319.01	26.6	25.0	1.6	1.07	mild
	2480	94	10	1911.66	32.3	25.3	7.1	1.28	moderate
	2490	57	10	3936.39	7.4	25.4	-18.0	0.29	–
	2500	81	50	2514.11	25.3	25.5	-0.2	0.99	–
	2550	94.3	15	1898.77	34.1	26.0	8.1	1.31	moderate
	2565	91	19	2042.94	32.6	26.1	6.5	1.25	moderate
	2584	81	22	2514.11	27.2	26.3	0.9	1.03	mild
	2606	61	20	3661.88	13.4	26.6	-13.1	0.50	–
	2626	106	24	1425.38	41.7	26.8	14.9	1.56	high
	2650	75	25	2825.61	24.8	27.0	-2.2	0.92	–
	2675	62	31	3596.06	15.8	27.3	-11.5	0.58	–
	2706	70	6	3104.86	22.6	27.6	-5.0	0.82	–
	2712	100	10	1661.22	40.7	27.6	13.1	1.47	moderate
	2722	57	6	3936.39	12.6	27.7	-15.1	0.46	–
	2728	106	17	1425.38	44.0	27.8	16.2	1.58	high
	2745	72	60	2990.84	24.9	28.0	-3.1	0.89	–
Tiemulike Formation P_2_t	2805	97	23	1784.51	41.3	28.6	12.7	1.44	moderate
	2828	108	22	1349.73	47.2	28.8	18.4	1.64	high
	2850	72	12	2990.84	27.3	29.0	-1.8	0.94	–
	2862	71	12	3047.44	26.9	29.2	-2.3	0.92	–
	2874	89	8	2132.89	38.5	29.3	9.2	1.31	moderate
	2882	70	21	3104.86	26.6	29.4	-2.8	0.91	–
	2903	100	10	1661.22	45.0	29.6	15.5	1.52	high
	2913	123	2	823.34	55.7	29.7	26.0	1.88	very high
	2915	78.7	12	2630.70	33.2	29.7	3.5	1.12	mild
	2927	71.6	24	3013.38	28.8	29.8	-1.1	0.96	–
	2951	69	13	3163.09	27.4	30.1	-2.6	0.91	–
	2964	117	3	1025.75	54.3	30.2	24.1	1.80	high
	2967	67	28	3282.15	26.3	30.2	-3.9	0.87	-
	2995	66	20	3343.01	26.2	30.5	-4.3	0.86	-
	3015	76	7	2772.00	33.8	30.7	3.0	1.10	mild
	3022	128	6	662.06	60.2	30.8	29.4	1.95	very high
	3028	72	23	2990.84	31.3	30.9	0.5	1.01	mild
	3051	80	3	2564.39	37.2	31.1	6.1	1.19	mild
	3054	125	13	758.05	59.7	31.1	28.6	1.92	very high
	3067	100	27	1661.22	48.8	31.3	17.5	1.56	high
	3094	80	16	2564.39	38.1	31.5	6.6	1.21	moderate
	3110	87	9	2224.88	42.7	31.7	11.0	1.35	moderate
	3119	105	31	1463.75	52.4	31.8	20.6	1.65	high
	3150	72	35	2990.84	34.1	32.1	2.0	1.06	mild
	3185	71	15	3047.44	34.2	32.5	1.7	1.05	mild
	3200	84	25	2366.91	43.0	32.6	10.4	1.32	moderate
	3225	93	30	1954.95	48.7	32.9	15.8	1.48	moderate
	3255	93	35	1954.95	49.4	33.2	16.2	1.49	moderate
	3290	71	30	3047.44	36.6	33.5	3.0	1.09	mild
	3320	87	20	2224.88	47.5	33.8	13.6	1.40	moderate
	3340	89	25	2132.89	49.1	34.0	15.0	1.44	moderate
	3365	110	7	1275.46	60.3	34.3	26.0	1.76	high
	3372	102	2	1581.07	56.7	34.4	22.3	1.65	high
	3374	67	14	3282.15	35.5	34.4	1.1	1.03	mild
	3388	88	5	2178.63	49.6	34.5	15.1	1.44	moderate
	3393	59	7	3796.80	29.6	34.6	-5.0	0.85	–
	3400	90	7	2087.67	51.0	34.7	16.3	1.47	moderate
	3407	94	22	1911.66	53.3	34.7	18.6	1.54	high
	3429	90	26	2087.67	51.6	34.9	16.7	1.48	moderate
	3455	85	45	2319.01	49.4	35.2	14.1	1.40	moderate
	3500	80	26	2564.39	47.3	35.7	11.6	1.33	moderate
	3526	92	35	1998.71	54.9	35.9	19.0	1.53	high
	3561	70	32	3104.86	42.0	36.3	5.7	1.16	mild
	3593	73	41	2935.01	44.8	36.6	8.2	1.22	moderate
	3634	68	24	3222.18	42.2	37.0	5.1	1.14	mild
	3658	70	2	3104.86	44.2	37.3	6.9	1.18	mild
	3660	106	10	1425.38	65.1	37.3	27.8	1.75	high
	3670	50	13	4466.72	27.5	37.4	-9.9	0.73	–
	3683	70	25	3104.86	44.7	37.5	7.2	1.19	mild
	3708	72	25	2990.84	46.7	37.8	8.9	1.24	moderate
	3733	76	21	2772.00	50.0	38.0	12.0	1.31	moderate
	3754	73	24	2935.01	48.5	38.3	10.2	1.27	moderate
	3778	76	24	2772.00	51.0	38.5	12.5	1.33	moderate
	3802	64	–	3467.56	42.9	38.7	4.2	1.11	mild

### Distribution characteristics of abnormal pressure in drillings

**Fig. 5:**
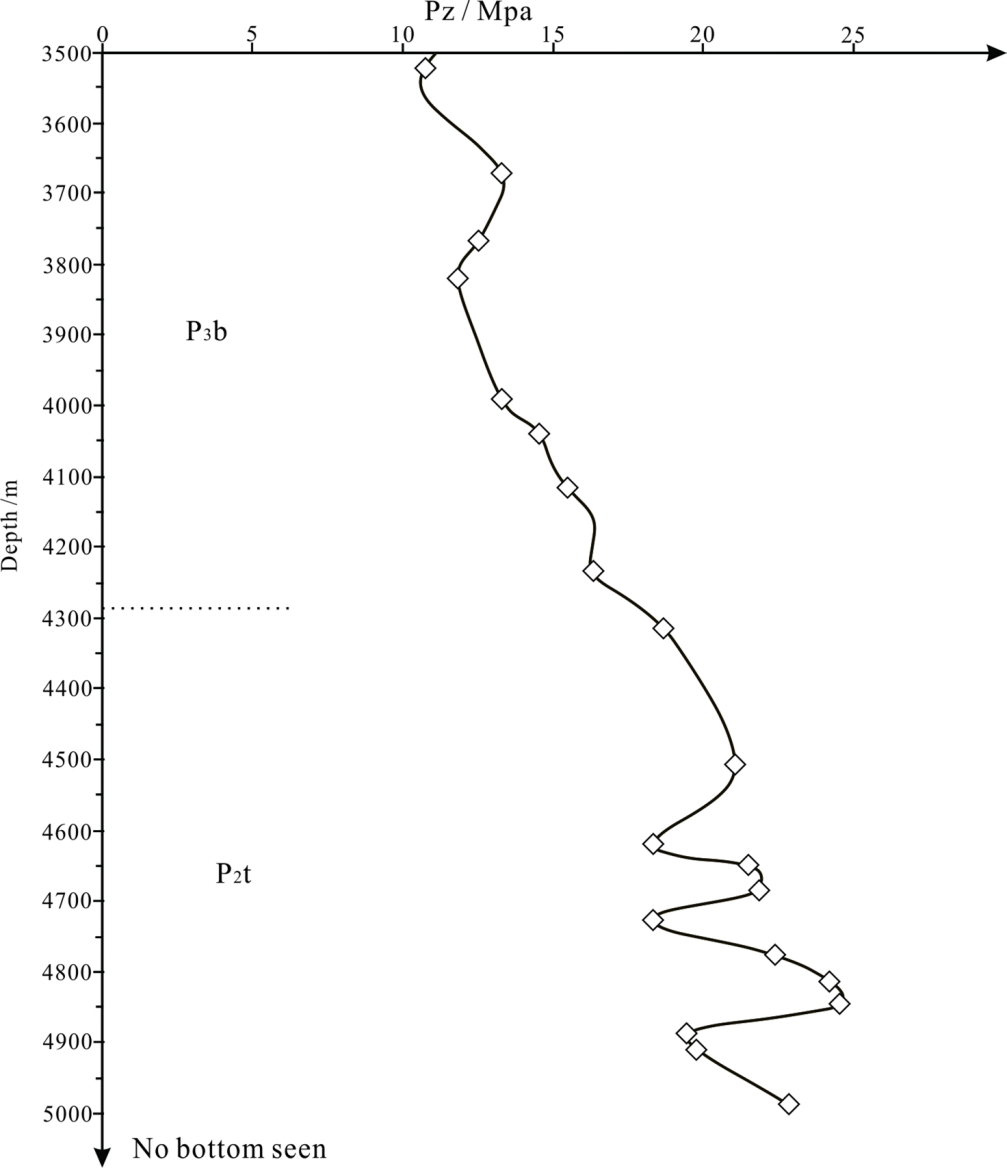
Curve of Abnormal Excess pressure with buried depth in Yican 1 well (this figure is generated in CorelDRAW 2018 software, https://www.coreldrawchina.com/).

Adopting the equilibrium depth method, the abnormal excess pressure of dark shale in the Tiemulike Formation was quantitatively calculated for the first time. Taking the abnormal excess pressure profile of Yican 1 well as an example, the excess pressure of the shale in the Tiemulike Formation ranges from 18.17 MPa to 24.60 MPa, with an average of 21.11 MPa, and the excess pressure gradually increased from the top to the deeper layers in the Tiemulike Formation. The abnormal excess pressure in Tiemulike Formation is about 5–10 MPa higher than that in the overlying Late Permian Basiergan Formation, indicating high abnormal pressure characteristics. There are three abnormal pressure significantly increased layers sections, forming fluid pressure compartment. The first significantly increased layer is about from 4650 to 4690 m in depth, the second significantly increased layer is about from 4750 to 4850 m in depth, and the third significantly increased layer is about from 4970 to 5010 m in depth of Yican 1well (Fig. 5).

### Distribution characteristics of abnormal pressure on the profile

According to calculations, the abnormal excess pressure in the shale of Tiemulike Formation in Yican 1 well, Ning 3 well, and Ning 4 well is relatively high. There is no abnormal excess pressure in the shale layers of Tiemulike Formation in Yi 2 well, due to later tectonic uplift, the early excess pressure was lost completely.

**Fig. 6:**
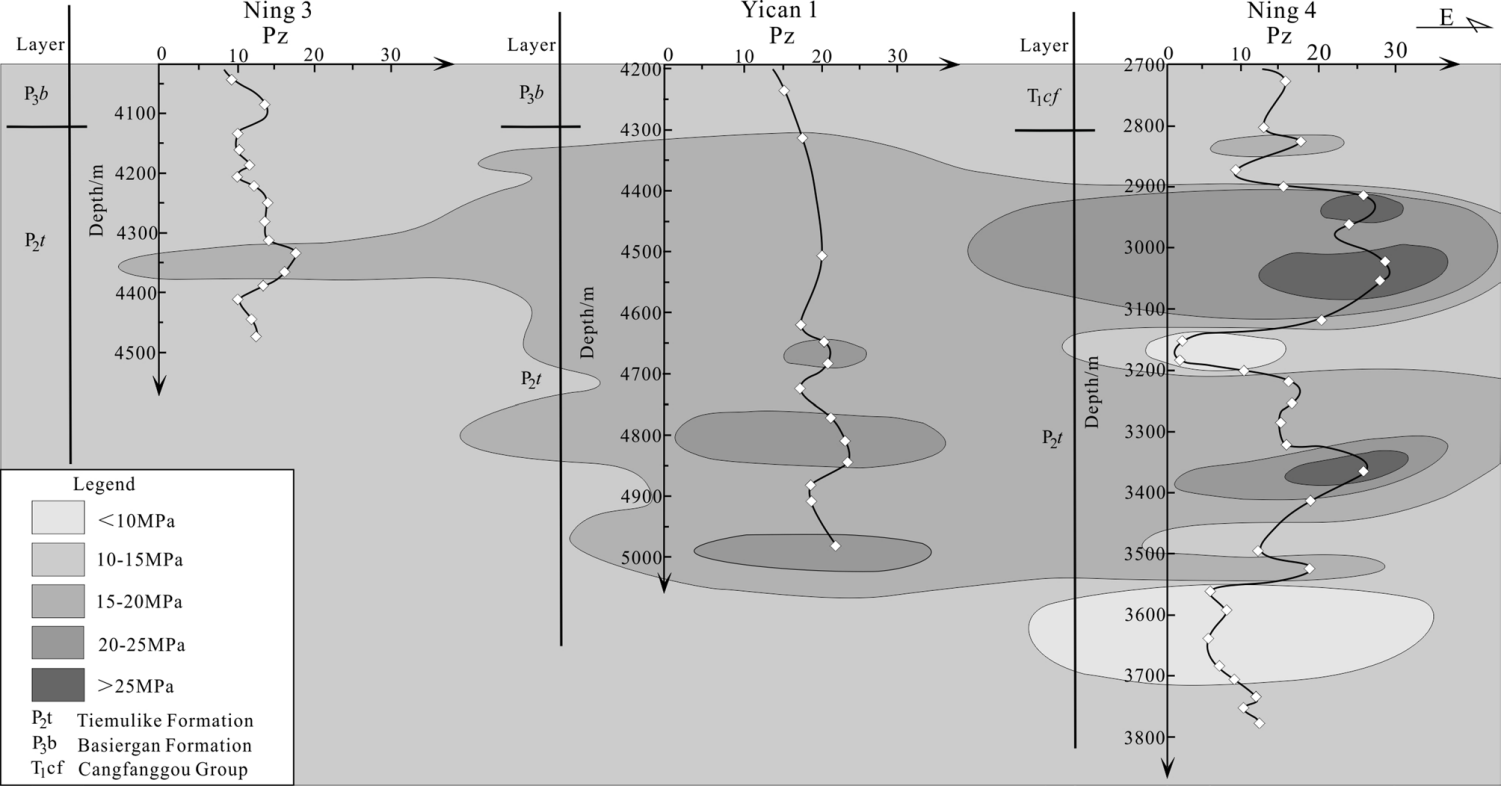
Abnormal Pressure Profile of Tiemulike Formation in the Central Low-lying zone of Yining Sag (this figure is generated in CorelDRAW 2018 software, https://www.coreldrawchina.com/).

In this study, the well-tie comparison profile of abnormal excess pressure developed in shale of Tiemulike Formation had been drawn, consists of Ning3 well, Yican 1 well, and Ning 4 well, which is extending near East to West in the central low-laying zone of the Yining Sag. From the well-tie comparison profile of abnormal excess pressure, we can see that the abnormal excess pressure is mostly above 10 MPa, up to 29.4Mpa. The abnormal excess pressure in Tiemulike Formation is about 5–10 MPa higher than that in the overlying Late Permian Basiergan Formation, indicating high abnormal pressure characteristics (Fig. 6). Because of thickened and pure dark mudstone layer with higher thermal evolution degree, several layers with sudden increased abnormal excess pressure appeared in vertical. There are three abnormal pressure significantly increased layers sections in Yican 1 well, Ning 4 well and Ning 3 well. The first significantly increased layer is about from 2905 to 2970 m in depth, the second significantly increased layer is about from 3010 to 3090 m in depth, and the third significantly increased layer is about from 3350 to 3410 m in depth of Ning 4 well. The first significantly increased layer is about from 4650 to 4690 m in depth, the second significantly increased layer is about from 4750 to 4850 m in depth, and the third significantly increased layer is about from 4970 to 4985 m in depth of Yican 1well. The first significantly increased layer is about from 4170 to 4190 m in depth, the second significantly increased layer is about from 4330 to 4380 m in depth, and the third significantly increased layer is about from 4450 to 4475 m in depth of Ning 3 well. The Higher abnormal excess pressure has good continuity in the horizontal direction, several high abnormal fluid pressure compartments were formed in the central low-laying area of Yining Sag, providing dynamic conditions for the expulsion of hydrocarbons from shale and the formation of shale gas reservoirs.

Unfortunately, there was no well drilled through the Tiemulike Formation and the underlying strata in the Yining Sag. Therefore, this study did not fully characterize the abnormal excess pressure situation of the Temurike Formation, especially the abnormal excess pressure characteristics in the lower part of the Tiemulike Formation are not clear.

## Disscussion

The overpressure section in the Yining Sag is developed in the thick and pure lithological layer of dark shale in the Tiemulike Formation. The response characteristics of logging curves from Yican 1 well, Ning 3 well, and Ning 4 well show that as the burial depth of the overpressure section increasing, the acoustic time difference is increasing, the velocity decreasing, and the density remains unchanged or slightly decreasing. The characteristics of logging curve reflect that the overpressure section in shale of Tiemuike Formation may be caused by the shale hydrocarbon generation.

**Fig. 7:**
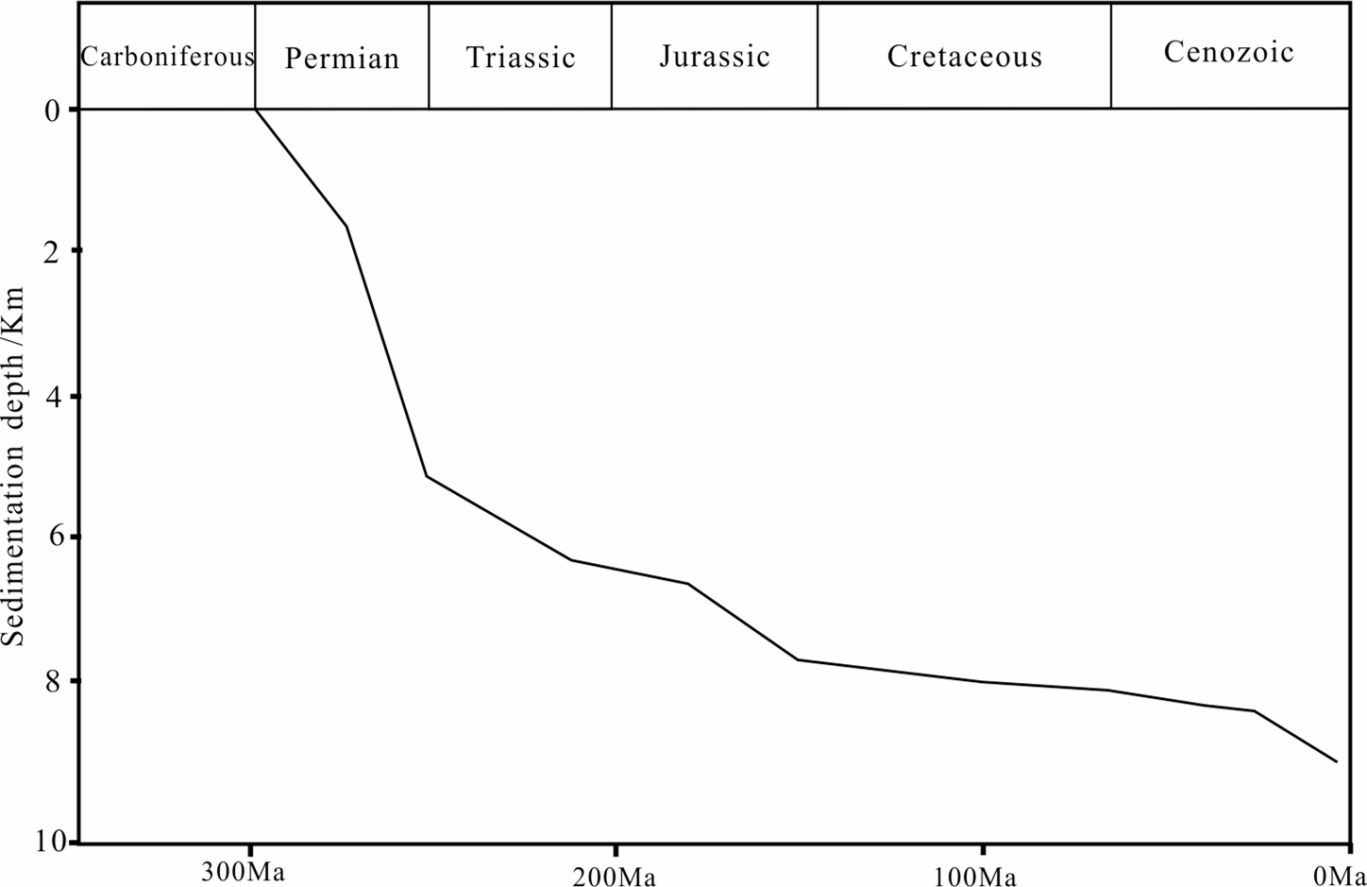
Curve of relationship between subsidence depth and geological time in Ili region (revised after^17,30,31^) (this figure is generated in CorelDRAW 2018 software, https://www.coreldrawchina.com/).

After the deposition of the Tiemulike Formation in Yining Sag, the sedimentation velocity was fast^17^, and the average subsidence rate during the Permian period was 139 m/Ma^30^. From the Early Triassic to the Late Jurassic, the Yining Sag continued to sink steadily, the subsidence rate was 30 m/Ma. The Middle Jurassic was another period of rapid subsidence in the Yining Sag, the subsidence rate was 73–202 m/Ma (Fig. 7)^17,30,31^. Due to the high subsidence rate, the dark shale of Tiemulike Formation entered the hydrocarbon generation threshold in the Late Permian. Because of Yining Movement, the Yining Sag upraised at the end of Permian. The duration of this tectonic event was very short, the lifting amplitude was very small, and the erosion amount in most areas of Yining Sag is between 40-60 m^26^.

**Fig. 8:**
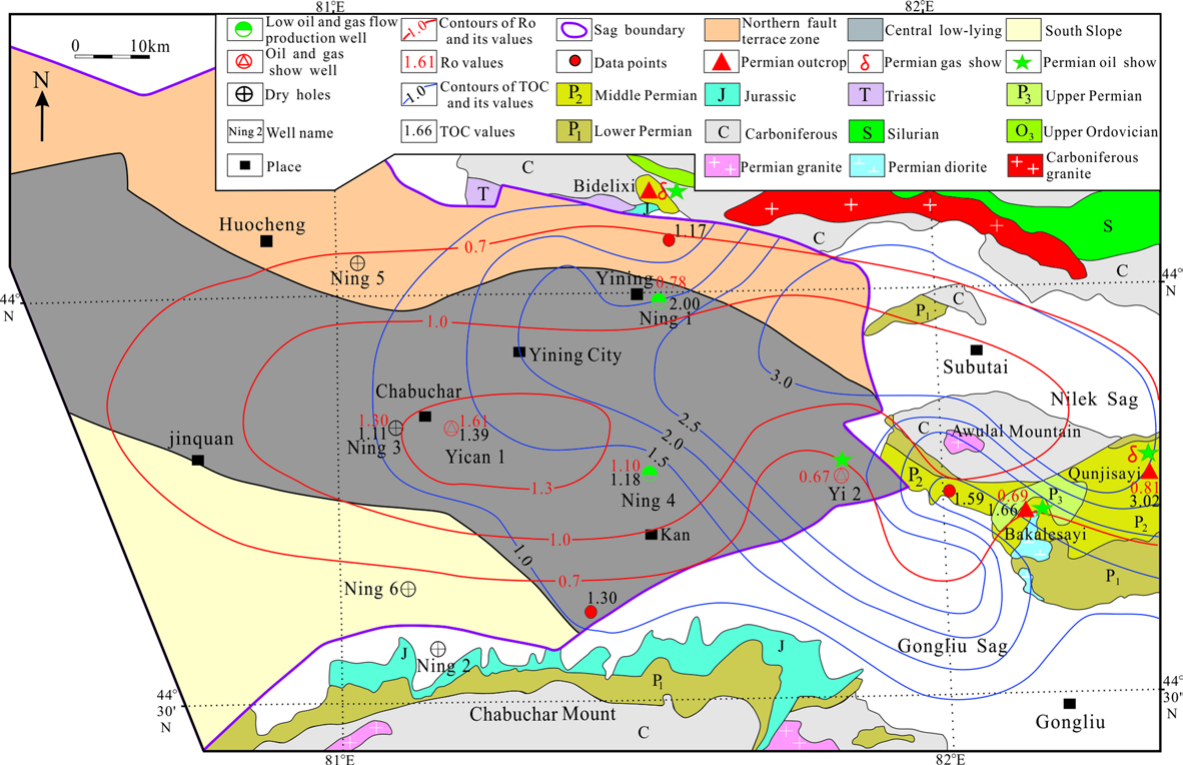
Superimposed distribution of TOC with Ro of the mud shale in Tiemulike Formation in Yining Sag area (this figure is generated in CorelDRAW 2018 software, https://www.coreldrawchina.com/).

The organic carbon (TOC) content ranges from 0.34 to 4.79%, with an average of 1.27%, data from 37 Black mud shale and marl samples in Tiemulike Formation in Yican 1 well. The organic carbon (TOC) content ranges from 0.80% to 2.33%, with an average of 1.18%, data from 10 Black mud shale samples in Tiemulike Formation in Ning 4 well. The organic carbon (TOC) content ranges from 0.36% to 2.37%, with an average of 1.11%, data from 33 Grey mud shale samples in Tiemulike Formation in Ning 3 well. We also test some samples of Tiemulike Formation in periphery outcrop of Yining Sag. The organic carbon (TOC) content ranges from 0.05% to 2.92%, with an average of 1.17%, data from 11 black mud shale samples in Tiemulike Formation in Bidelixi outcrop on the North of Yining Sag. The organic carbon (TOC) content ranges from 1.04% to 2.71%, with an average of 1.66%, data from 7 black mud shale samples in Tiemulike Formation in Bakalesayi outcrop on the East of Yining Sag. The organic carbon (TOC) content ranges from 0.64% to 2.91%, with an average of 1.34%, data from 7 dark gray marl samples in Tiemulike Formation in Bakalesayi outcrop on the East of Yining Sag. The abundance of TOC in the black shale tended to increase from the South to the North of the Yining Sag (Fig. 8). The kerogen types are classified into II_2_-III types.

From the microscopic components in the dark mud shale of the Tiemulike Formation, take Ning3 well as an example, the content of sapropel and chitin is relatively high. The content range of sapropel group is 0–97.3%, mostly ranging from 10 to 70%, with an average of about 40%. The content range of chitin is 0–81%, mostly between 20 and 40%, with an average of 30%. The content of vitrinite is 6–87.5%, mostly 20–30%, with an average of about 25%. The content of inertinite is 0–62.5%, mostly 10–25%, with an average of about 15%. These data indicate that the mud shale of the Tiemulike Formation has strong hydrocarbon generation potential.

**Fig. 9:**
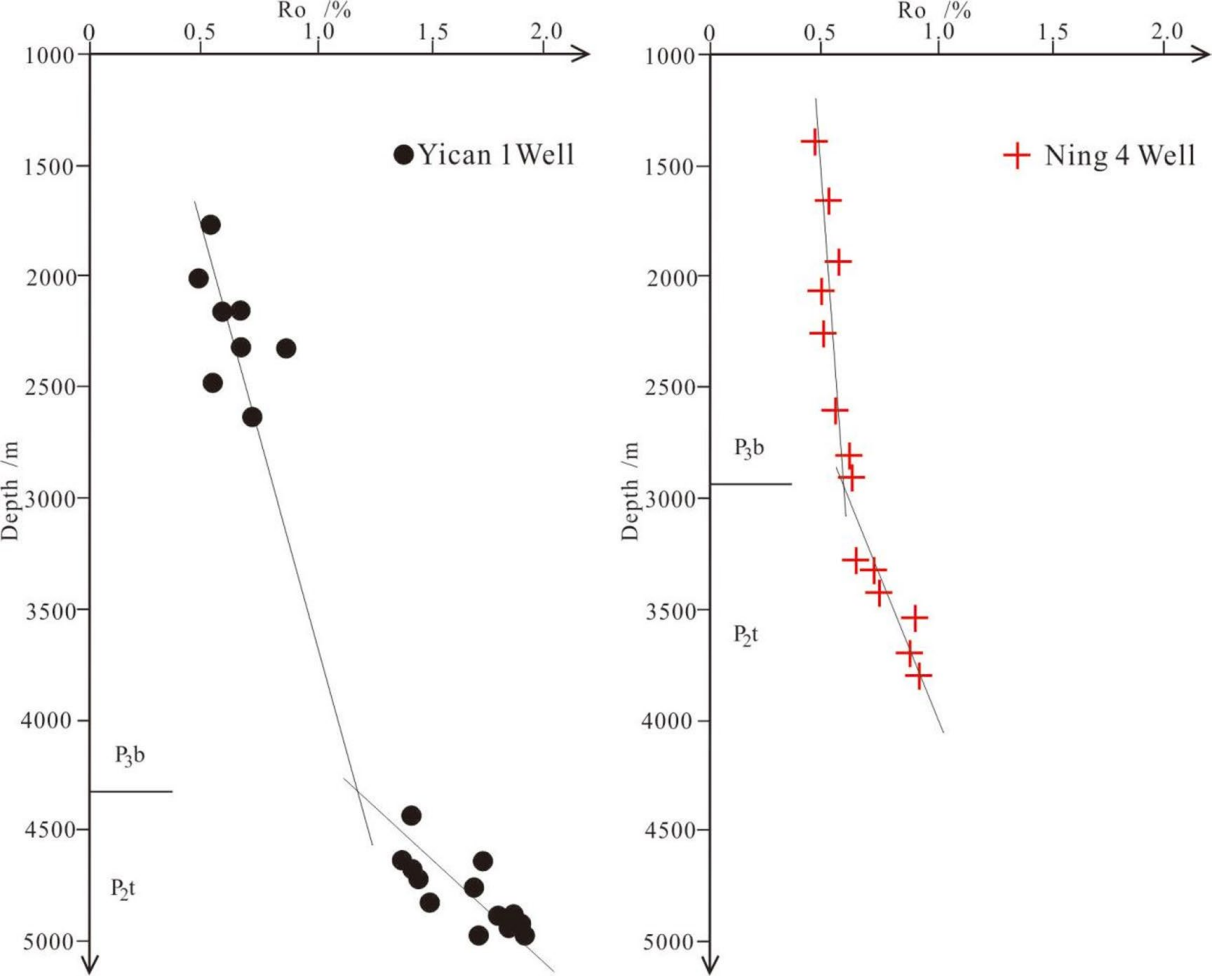
The vitrinite reflection(Ro) vs depth in Yican 1 well (left) and Ning 4 well (this figure is generated in CorelDRAW 2018 software, https://www.coreldrawchina.com/).

The value of Ro was approximately 0.8% at the edge, and the dark shale recently entered the mature stage in the source rock evolution process. The value of Ro mostly ranged from 1.0% to 1.5% in the central area. The weighted average value of Ro was 1.29%, and the dark shale entered a highly mature stage in the source rock evolution process. The results indicate that the dark mud shale of the Tiemulike Formation in the Central Low-lying zone of Yining Sag has mostly entered the high maturity stage, the dark mud shale of the Tiemulike Formation in the northern part of the Yining sag is in the mature stage, the maturity of the dark mud shale evolution in the Tiemulike Formation in the eastern part of the Yining sag is the lowest, and most of them are still in the low maturity stage (Fig. 8). The vitrinite reflection(*Ro*) vs depth have been studied, there is a turning point in the data fitting line of Yican 1 well and Ning 3 well, which is belonging to a two-part model, showing that Ili Basin has the characteristic of "hot in ancient times and cold now". It is consistent with the depth of the abnormally high pressure, the depth the super-pressure is going to form is about 4300 m in Yican 1 well, and 2900 m in Ning 4 well (Fig. 9).

**Fig. 10:**
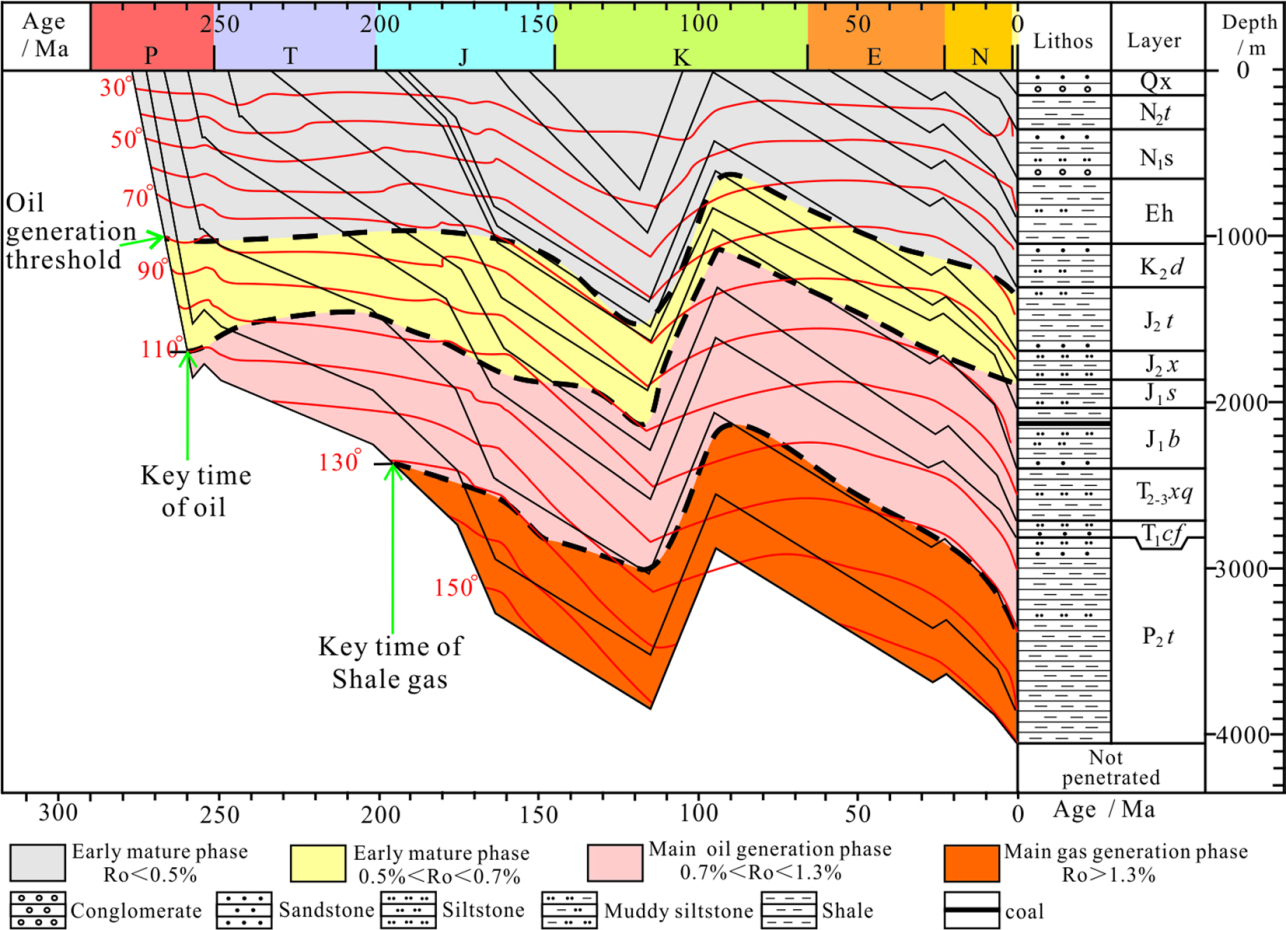
Burial history of Tiemulike Formation in Ning 4 well of the Yining sag (this figure is generated in CorelDRAW 2018 software, https://www.coreldrawchina.com/).

The burial history of Yining Sag has been studied. The research results indicate that Yining sag had experienced mainly four subsidence and erosion stages since Permian (Fig. 10). The maximal sedimentation rate existed from middle Permain to early Triassic, and Yining sag uplifted in a short time in late Permain. The Yining sag quickly subsided from early Triassic to late Cretaceous, in the late Early Jurassic, the shale of the Tiemulike Formation began to enter the gas generation stage. During the Middle to Late Jurassic, the temperature at the bottom of the Tiemulike Formation reached 150℃, the thermal evolution of shale in the Tiemulike Formation reached a high level, causing a further rapid increase of the pressure in the shale of Tiemulike Formation^17,31^.

Yining sag had a large scale uplift and erosion in early Cretaceous, and sedimentation rate decreased from early stage of late Cretaceous to the end of Paleogene, in which basin quickly uplift caused by the effect from Himalayan movement. The sag slowly deposited from early Neogene to late Pleistocene, and it is in the erosion condition nowadays. Although there was a significant uplift process in the end of Early Cretaceous, however, the Tiemulike Formation was still within the hydrocarbon generation threshold, and the process of hydrocarbon generation and pressurization had been ongoing. The shale in Tiemulike Formation had been deeply buried and experienced a medium to high temperature environment (80℃-150℃), the thermal evolution degree of organic matter in dark shale was relatively high. Due to the generation of hydrocarbons from organic matter in shale of Tiemulike Formation, the volume expansion ratio can reach 3–7%, 7–8 times the porosity of the shale itself.

Yi 2 well is located on the eastern edge of Yining Sag, the bury depth of shale in Tiemulike Formation is shallow at present^32^. After calculation, there was no abnormal excess pressure in shale of Tiemulike Formation. It is believed that the shale of the Tiemulike Formation in Yi 2 well may not have undergone the process of deep burial for hydrocarbon generation. Due to hydrocarbon generation in the dark shale of the Tiemulike Formation, several high abnormal fluid pressure compartments were formed in the central low-laying area of Yining Sag. The Yi 2 well is located in the atmospheric pressure area, which is one of the target areas for shale oil and gas migration. Multiple shale oil and gas indications such as oil spots and fluorescence can be seen in Yi 2 well, which partly proved the increase in pressure caused by hydrocarbon generation from dark shale in the central low-laying area of Yining Sag.

On the other hand, it was found that the transformation of Montmorillonite into Illite was particularly developed during the diagenesis of shale in the Tiemulike Formation^17^. According to study conducted by Powers (1967), during the process of Montmorillonite converting into Illite, the multi-layered single-molecule water inside the Montmorillonite crystal structure had been released, becoming intergranular free water^33^. The main diagenetic reactions are shown in Eq. (8). The density of multi-layered single-molecule water is approximately 1.4 g/cm^3^, while the density of free water is 1.0 g/cm^3^. When the transformation of Montmorillonite converting into Illite occurs, the volume of fluid in shale micro pore-fracture expands, causing an increase of pressure in shale layers.

8 $${\mathrm{Montmorillonite}} + K - {\mathrm{feldspar}} + {\mathrm{Muscovite}} \to {\mathrm{Illite}} + {\mathrm{Chlorite}} + {\mathrm{H}}_{2} {\mathrm{O}} + {\mathrm{Quartz}}$$

The abnormal excess pressure is widely developed in shale of sedimentary basins, the overpressure caused by hydrocarbon generation and non-equilibrium compaction on a global scale accounts for about 92.5%^3,16,31,34–36^. The influencing factors of abnormal pressure in Ili basin are indeed diverse, such as hydrocarbon generation, clay mineral diagenesis, disequilibrium compaction, tectonic compression, and so on. According to analogical analysis on logging response characteristics of overpressure layers at home and abroad, combining the sedimentary environment of the shale in Tiemulike Formation^18^ , burial history & hydrocarbon generation history, etc^17,31^, we found that the formation of abnormal excess pressure in the shale of the Tiemulike Formation in the Yining Sag was mainly controlled by the hydrocarbon generation expansion of dark shale and clay mineral diagenesis. The fault zones or lithofacies changes (e.g., carbonate interbeds) might disrupt horizontal continuity of high-pressure compartments beyond the studied area, there maybe is a lateral heterogeneity risk of abnormal pressure.

## Conclusion

1. There are several weak high pressure to Ultra-high pressure layers in shale of Tiemulike Formation, with the pressure coefficient range from 1.21 to 1.95. The abnormal excess pressure distribution profile in single well shown that the excess pressure gradually increased from the top to the deeper layers in the Tiemulike Formation, with three abnormal pressure significantly increased layers. The Higher abnormal excess pressure has good continuity in the horizontal direction, several high abnormal fluid pressure compartments were formed in the central low-laying area of Yining Sag.
2. The cause of abnormal excess pressure in the shale of Tiemulike Formation in Yining Sag belongs to composite genesis, which was mainly controlled by the hydrocarbon generation and expansion of deeply buried dark shale, and also influenced by converting process of Montmorillonite into Illite in the diagenesis of clay minerals.

## Data Availability

The data are available from the corresponding author upon reasonable request.
